# Targeting androgen receptor (AR) with a synthetic peptide increases apoptosis in triple negative breast cancer and AR‐expressing prostate cancer cell lines

**DOI:** 10.1002/cnr2.1922

**Published:** 2023-10-30

**Authors:** Mazdak Jamshidi, Fatemeh Keshavarzi, Sabrieh Amini, Ismail Laher, Ali Gheysarzadeh, Kambiz Davari

**Affiliations:** ^1^ Department of Biology, Sanandaj Branch Islamic Azad University Sanandaj Iran; ^2^ Department of Anesthesiology, Pharmacology and Therapeutics The University of British Columbia Vancouver British Columbia Canada; ^3^ Department of Clinical Biochemistry Ilam University of Medical Sciences Ilam Iran

**Keywords:** androgen receptor, anticancer peptide, apoptosis, breast cancer, dihydrotestosterone (DHT)

## Abstract

**Background:**

The androgen receptor (AR) has been studied as an approach to cancer therapy.

**Aims:**

We used human breast cancer‐derived cells with high, low, and very low expression levels of AR, in addition to prostate cancer‐derived LNCaP and DU‐145 cells as a positive and negative controls to examine apoptosis caused by a synthetic peptide that targets ARs.

**Methods and Results:**

The peptide was produced to inhibit AR transactivation in breast cancer cell lines. We then measured cell viability, caspase‐3 activity, and the ratio of Bax/Bcl‐2. The findings indicated that the peptide (100–500 nM) in the presence of dihydrotestosterone (DHT) reduced cell growth in cells with high and low expression level of AR (*p* < .001), but not in cells with very low levels of AR. Treatment with 100–500 nM of peptide activated caspase‐3 and increased the ratio of Bax/Bcl‐2 in cells with high and low expression levels of AR. Also, increasing concentrations of the peptide (100–500 nM) reduced BrdU incorporation in the presence of DHT and promoted apoptosis in cells with high and low expression levels of AR (*p* < .001).

**Conclusion:**

The findings indicate the peptide significantly increased apoptosis in cancer cells.

## 1 INTRODUCTION

Triple‐negative breast cancer (TNBC) is an aggressive type of cancer, representing approximately 10% of all breast cancers^1^, ^2^ that is characterized by the absence of expression of estrogen receptors (ERs), progesterone receptors (PRs), and epidermal growth factor receptor 2 (HER‐2).^3^ TNBC accounts for a significant percentage of deaths among breast cancer patients, with a 5‐year survival rate of approximately 83% compared to 89%–96% for non‐TNBC.^4^, ^5^ A growing body of evidence suggests that the androgen receptor (AR) may be a promising target for therapy, as its expression is associated with a poor prognosis in TNBC.^6^ Structural analysis of AR indicates roles both as a factor in nuclear transcription and a receptor for steroid hormones.^7^ The AR contains as is the case for other steroid receptors: a ligand‐binding domain (LBD) that is smaller than in other steroid receptors, two DNA‐binding domains (DBD) linked by a third domain, and a poorly conserved region in its N‐terminal domain (NTD). When ligands such as testosterone and dihydrotestosterone (DHT) attach to the LBD, ARs dissociate from chaperone proteins, allowing for AR dimerization and activation of nuclear androgen response elements.^8^, ^9^, ^10^ Targeting the AR has been studied as a potential cancer therapy, but with limited efficacy due to drug resistance.^11^

Peptides are small compounds composed of protein macromolecules, consisting of a chain of less than 50 amino acids (AA). They can be produced through bioactive or natural peptides, engineered peptides, and peptides derived from chemical libraries. Low molecular weight peptides have a powerful ability to penetrate tumor tissues. Anti‐cancer peptides can be derived from plants and animals, but they can also be chemically synthesized, providing a cost‐effective cancer treatment.^12^, ^13^, ^14^ These peptides work by targeting abnormal intracellular signaling pathways or cell entry.^15^

Peptide derivatives, which are designed to compete with tumor‐localizing peptides that specifically bind to receptors on the surface of cancer cells, have the potential to enhance tumor therapy. These peptides work by inhibiting oncogenic signaling pathways that regulate cancer cell activity, prevent apoptosis, and promote tumor cell proliferation.^16^

Peptide therapy has emerged as a promising treatment for cancer, particularly in breast cancer where the cells become more resistant to traditional drug therapies.^17^ Some research groups Drager et al.^18^ and Bidwell et al.^19^ have developed peptides that can inhibit the binding c‐Myc to DNA. Additionally, Zhao et al.^20^ explored the use of peptides as cytolytic agents in cancer cells.^21^ The use of tumor suppressor proteins, which are synthesized peptides that regulate CDKs, is another therapeutic peptide in breast cancer.^22^ The peptides derived from p21 have prevent cell proliferation.^16^ Zhang et al. utilized these derived peptides to induce cell death in breast cancer cells.^23^ Estrogen receptor α (ERα) levels in breast cancer cells can be targeted for peptide‐based cancer therapy. In this regard, Dai et al. designed a compound that achieved an IC_50_ of approximately 9.7 μM by inducing ERα degradation.^24^ Furthermore, some peptides have been used to induce apoptosis of cancer cells by targeting the Bcl2 protein.^25^

Some modified peptides that affect the non‐transcriptional or transcriptional responses mediated by AR in various contexts indicate that a newly synthesized peptide with the sequence Gln‐Pro‐Lys‐His‐Phe‐Thr‐Glu‐Leu‐Tyr‐Phe‐Lys‐Ser interacts better with the transactivation motif of AR than other peptides.^26^ This peptide was initially chosen among 1012 random 12‐amino acid peptides by Lung Hsu and colleagues,^26^ and a later study by de Wijngaart and coworkers demonstrated that this peptide inhibits AR function.^27^, ^28^ Furthermore, this peptide contains the FxxLF finger motif. A study by Trampan et al. reported that the AR LBD serves as docking site for FxxLF motifs and that the cofactor gelsolin interacts with AR through an FxxFF motif.^27^, ^28^ These findings highlight the potential of a peptide inhibitor in combating breast cancer and offer new avenues for further research. This study assessed the potential anti‐cancer effects of this peptide in cancer cell lines.

## 2 MATERIALS AND METHODS

### 2.1 Solid‐phase peptide synthesis

**FIGURE 1:**
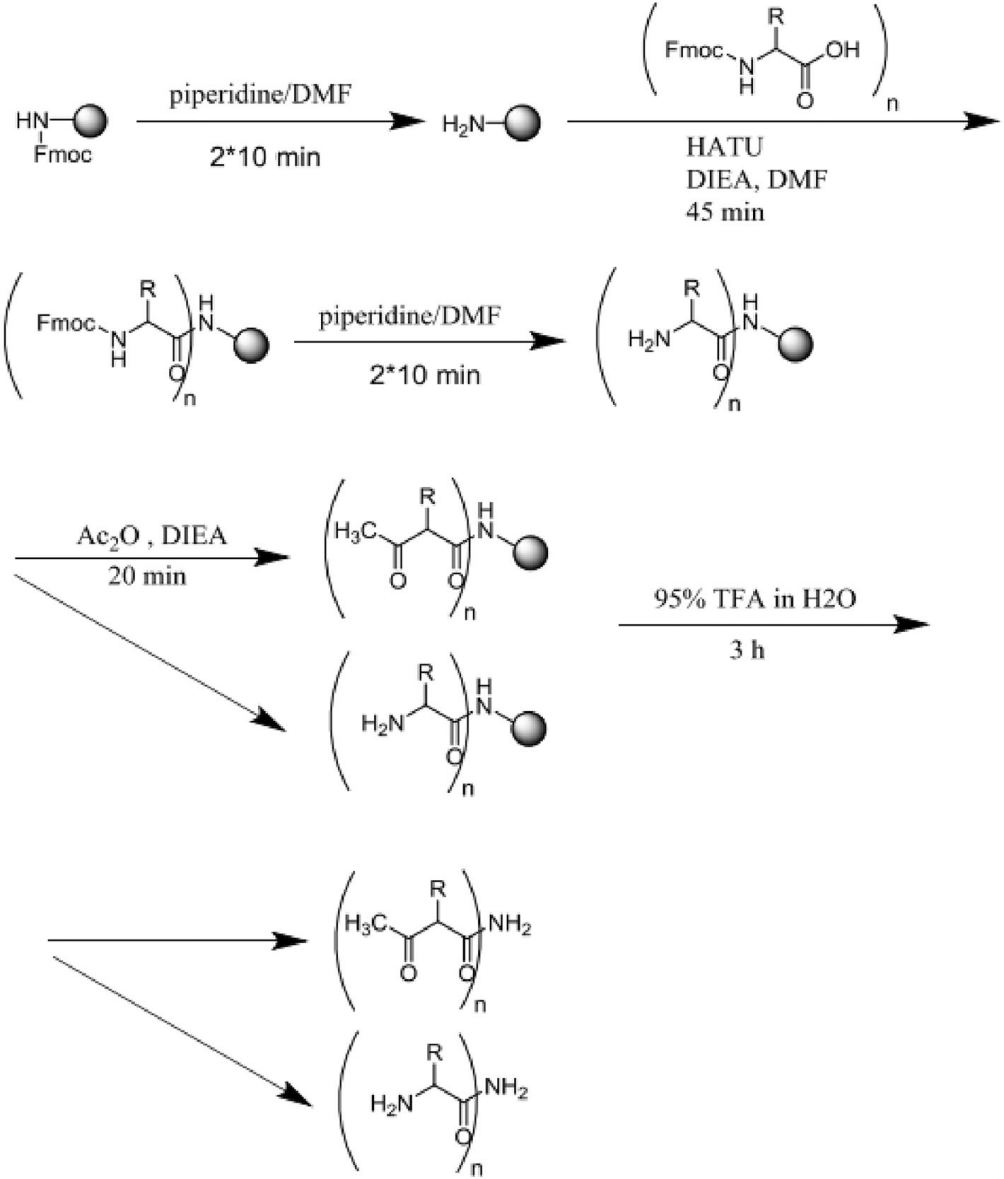
Flow chart of solid‐based peptide synthesis steps.

The innovative peptide (Gln‐Pro‐Lys‐His‐Phe‐Thr‐Glu‐Leu‐Tyr‐Phe‐Lys‐Ser) and a negative control peptide (Met‐Pro‐Phe‐Ser‐Tyr‐Gly‐Lys‐Arg‐Lys‐Lys‐Trp‐Lys‐Met‐Arg) (MPF) were synthesized using solid phase methods.^29^, ^30^ Briefly, standard analysis using a fluorenyl methoxycarbonyl (Fmoc) protecting group was performed at scale of 100 μmol L‐amino acids of the peptide and MPF on glass syringes (Figure 1).

#### 2.1.1 Wang resin as the solid phase platform

The resin was placed inside the reaction container with dichloromethane for 21 min. The dichloromethane was then removed from the reaction container using a vacuum pump, and 1.5 mL of dimethylformamide was added to the resin five times for 30 s each time. Finally, a vacuum pump was used to remove the solvent from the resin, completing the preparation for deprotection of the amine group.

#### 2.1.2 Deprotection of the amine group

The amine group of the first AA attached to the Wang resin and the amine group of the rinkamide resin were protected by Fmoc. To remove Fmoc, a solution of piperidine (21%) in dimethylformamide was added to the resin in two steps each lasting 11 min. After the reaction, the resin was washed five times for 31 s per wash cycle with dimethylformamide, and finally the surface of the resin was washed twice for 30 s each time with dichloromethane to ensure the removal of the protective group. The presence of a type 1 free amine group and the removal of the Fmoc group was indicated by the blue color obtained from the ninhydrin test.

#### 2.1.3 Connecting the first AA to the resin

The first AA of each peptide was placed in a test tube containing the HATU coupling agent and activator. To separate them from dimethylformamide (three equivalents from each peptide), they were completely dissolved using an ultrasonic bath. After obtaining a homogeneous solution, six equivalents of diisopropylethylamine (DIEA) resin were added to the solution, and the resulting mixture was then added to the resin and gently stirred for 45 min to initiate the reaction. At the end of the reaction, the solvent and additional materials were removed from the surface of the resin using a vacuum pump. The resin was then treated five times for 30 s each time with 1.5 mL of dimethylformamide and twice for 30 s each time with 1.5 mL of dichloromethane. To remove all remaining reagents, the resin was then washed. After the resin was dried with a vacuum pump, a ninhydrin test was performed to ensure binding of the AA to the resin. A yellow color indicated that there were no free amine group and the reaction was complete. After the ninhydrin test, the resin was washed again with dimethylformamide solvent to prepare for the removal of the Fmoc group of the AAs.

#### 2.1.4 Connecting the second AA and later AAs to the resin

After releasing the amine group in the first AA, subsequent AAs were added to the peptide sequence according to the procedures described above.

#### 2.1.5 Separating the peptide from the resin

Peptides were stirred with *trifluoroacetic acid* solution (95%) in water for 3 h. After separating the peptide from the resin, the solvent was evaporated using nitrogen gas (N_2_). Diethyl ether was then added to the peptide, and the remaining ether in the peptide precipitate was separated as overflow after centrifugation. This step of washing with ether was repeated five times and finally the remaining sediment was dissolved in water and acetonitrile and placed in a freeze dryer. The peptides produced were characterized using high‐performance liquid chromatography (gradient from 0% to 100% MeCN for 20 min, C18 column) and a purity of more than 95% was accepted. The synthesized peptides were then dissolved in sterile phosphate‐buffered saline (PBS) at the defined concentrations for the experiments according to previous studies.^29^, ^30^, ^31^, ^32^, ^33^, ^34^

### 2.2 Cell culture

Five cell lines with high (HTB‐131), low (HTB‐26), and very low (HTB‐132) expression levels of AR, as well as prostate cancer‐derived LNCaP and DU‐145 cells (as positive and negative controls)^31^, ^32^ were used. All cells were obtained from the Pasture Cell Bank (Tehran, Iran) and kept at 37°C in a humidified 5% CO_2_ atmosphere. Cells were cultured in Dulbecco's modified Eagle medium (DMEM) supplemented with 10% fetal bovine serum (FBS), penicillin (100 U/mL), streptomycin (100 U/mL), and glutamine (2 mM). Cells were sub‐cultured when they reached 70% confluence.

### 2.3 MTT assay

Cell proliferation of HTB‐131, HTB‐26, HTB‐132, LNCaP, and DU‐145 cells was evaluated with a 3‐(4,5‐dimethyl‐2‐thiazolyl)‐2,5‐diphenyl‐2‐H‐tetrazolium bromide (MTT) colorimetric assay (Sigma Aldrich, St. Louis, MO, USA). In brief, the cells (5 × 10^3^ cells/well) were cultured in DMEM for 24 h at 37°C. The cells were then treated with various concentrations of the peptides, and 10 μL of a 5 mg/mL MTT solution was added to each well and incubated for a further 4 h. To dissolve the brightly colored formazan crystals, 100 μL of DMSO was added, and the plates were incubated for 1 h at 37°C. The absorbance was then evaluated at 570 nm using a spectrophotometer (Biotech, Winooski, VT, USA).

### 2.4 Caspases‐3 activity assay

A colorimetric assay was used according to the manufacturer's instructions (Bio Vision, Winooski, VT, USA) to measure the activity of caspase 3. In brief, cells were cultured overnight and incubated with different concentrations of peptides for 24 h. Cells were then lysed on ice followed by centrifugation at 5000 rpm and the supernatants incubated with the caspase‐3 substrate. After 1 h of incubation at 37°C, the concentration of p‐nitroaniline was measured at 405 nm using a microplate reader (Biotech, Winooski, VT, USA).

### 2.5 Quantitative real‐time polymerase chain reaction (PCR)

The expression levels of Bax and Bcl‐2 genes were measured by real‐time (RT)‐ PCR. In brief, cells were treated with various concentrations of peptides for 24 h in a complete growth medium. Total RNA was extracted using an RNX‐Plus Kit and cDNA was synthesized using the Takara Transcription Kit (Takara Bio Inc., Tokyo, Japan) according to the manufacturer's instructions. A total of 2 μL of cDNA template, 1.5 μL each of forward and reverse primers and QuanTitect SYBR Green Master Mix were used to perform quantitative RT‐PCR. This method was performed using the following procedures: denaturation at 95°C for 15 min and 40 cycles of 94°C for 25 s and annealing temperature for 15 s. The sequences of the primers were as follows: BCL‐2 forward, 5́ cgacttcgccgagatgtccagccag 3́; BCL‐2 reverse, 5́ acttgtggcccagataggcacccag 3́; Bax forward, 5́ agggtttcatccaggatcgagcag 3́; Bax reverse, 5́ atcttcttccagatggtgagcgag 3́; β‐actin forward, 5́ tcatgaagatcctcaccgag 3́; and β‐actin reverse, 5́ ttgccaatggtgatgacctg 3́. Finally, the analysis was based on the β‐actin gene using 2∆∆CT.

### 2.6 Western blotting

Equal amounts of proteins from all cell lysates were loaded into wells of SDS‐PAGE followed by electrophoresis and the proteins were then transferred to PVDF membranes. These membranes were blocked for 1 h with 5% fetal bovine albumin at room temperature, and blotted with primary antibodies against AR and β‐tubulin (Cell Signaling, Washington, USA) for 2 h at 37°C. The membranes were then washed with buffer and incubated with HRP‐conjugated secondary antibodies for 1 h at room temperature. Finally, immune complexes were evaluated using electrochemical luminescence.

### 2.7 BrdU cell proliferation assay

Cells (density of 5 × 10^3^/well) were cultured for 24 h and then treated with 5‐bromo‐2′‐deoxyuridine (BrdU) followed by centrifugation at 5000 rpm for 5 min. The supernatants were discarded and a BrdU assay was performed. The cells were then incubated in BrdU labeling solution for 4 h at 37°C according to the manufacturer's instructions. Finally, the absorbance of the samples was measured at 450 nm using a plate reader.

### 2.8 Apoptosis detection by flow cytometry

We used the annexin V/propidium iodide double staining assay to evaluate apoptosis induced by the peptide. In brief, cells were seeded at a density of 2 × 10^4^ cells/well and then treated with various concentrations of peptides after 24 h. The cells were carefully harvested with a cell scraper and washed with PBS. Cells were then stained and the number of cells in each quadrant was counted.

### 2.9 Statistical analysis

All experiments were performed in triplicate with the results reported as the mean ± standard deviation (SD). The values in treated cells were calculated as a percentage of the values in untreated cells. Statistical analyses were done using version 20 of SPSS software (Chicago, IL, USA). Additionally, the flow cytometry data was analyzed using FC Salyzer software.

## 3 RESULTS

### 3.1 The cytotoxic activity of the peptide against human TNBC cell lines in vitro

**FIGURE 2:**
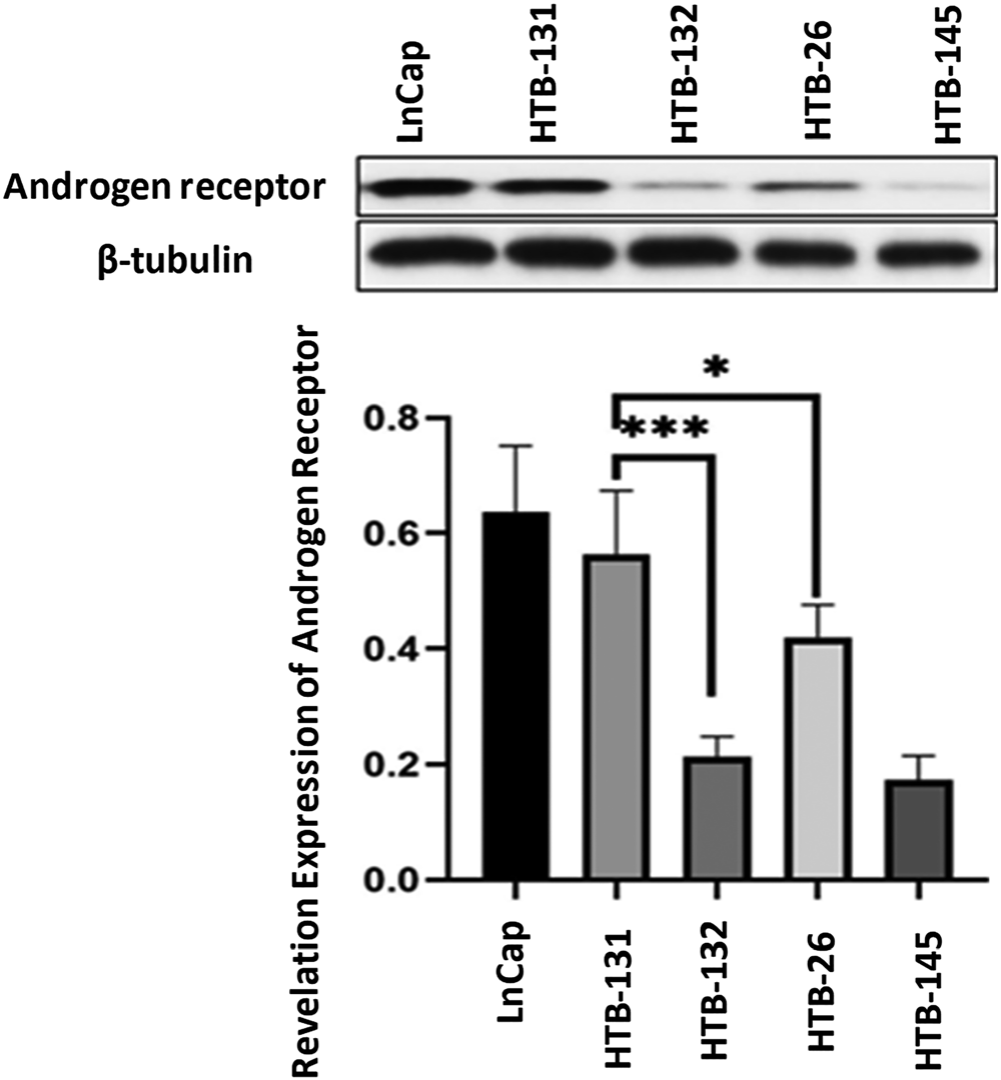
The proliferative effect of DHT on protein expression levels of the HTB‐131, HTB‐132, HTB‐26, LNCaP, and DU‐145 cell lines. The androgen receptor (AR) expression was quantified based on beta‐tubulin. The results were obtained from three independent experiments. The AR expression levels were compared in breast cell lines using one‐way ANOVA, *p < .05; ***p < .001.

The cytotoxic effects of the peptide were evaluated using an MTT assay, where the proliferative effect of DHT on AR‐expressing cells was measured. Cells with high expression levels of AR (HTB‐131 cells) were more sensitive to DHT compared to cells with low levels of expression of AR (HTB‐132 cells) (Figure 2).

**FIGURE 3:**
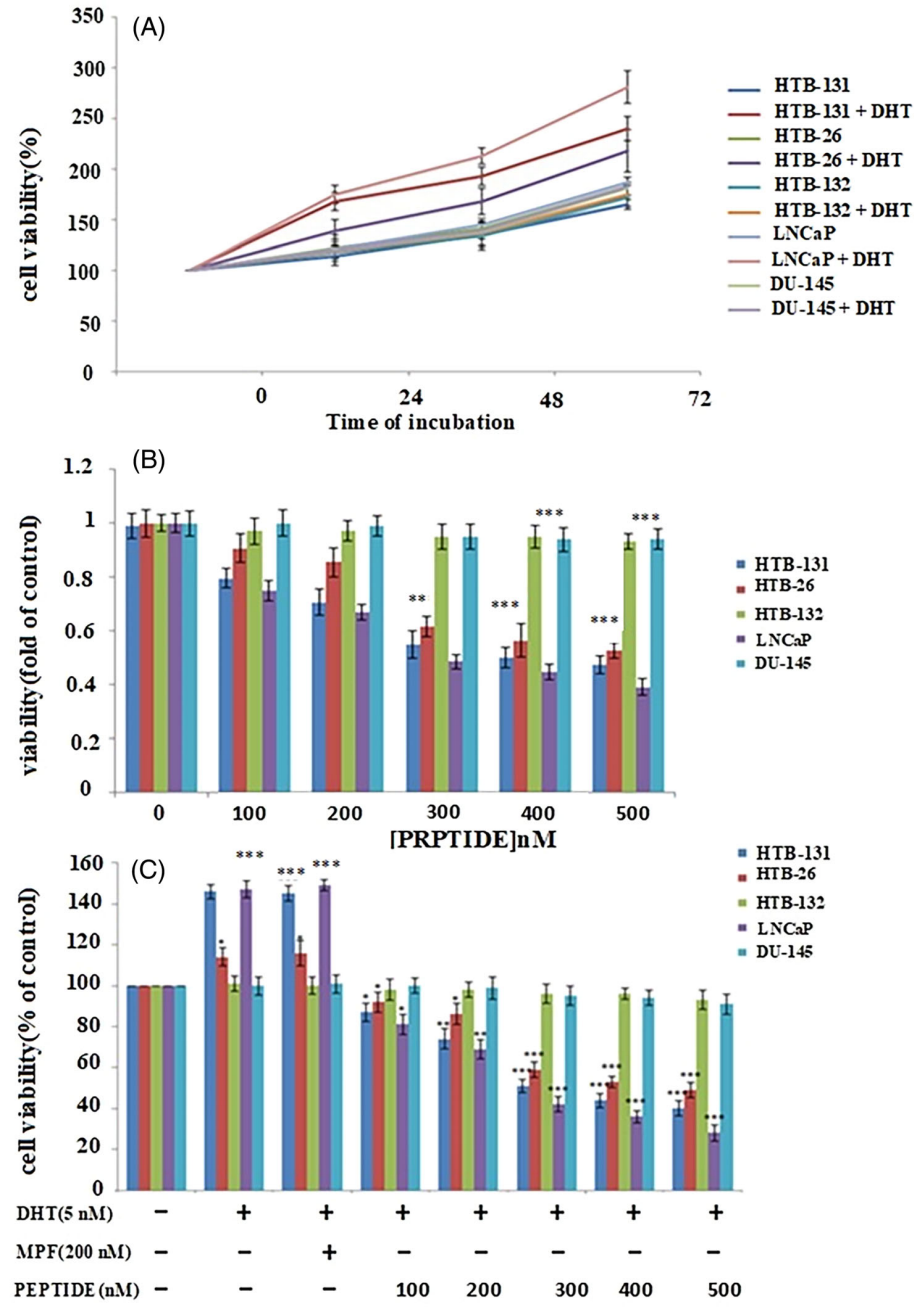
Cytotoxic effects of the peptide in androgen‐expressing cells. (A) Cells were treated with different concentrations of the peptide for 24, 48, and 72 h in the presence and absence of dihydrotestosterone (DHT) and cytotoxicity was assessed using a MTT assay. (B) The peptide treatment alone without DHT and (C) The peptide reduced cell viability of androgen receptor‐expressing cells in a dose‐dependent manner. Results (mean ± SD) were calculated as a percent of control values. Statistical analysis by one‐way ANOVA. *p < .05, **p < .01, and ***p < .001 indicates significant differences.

High (LNCaP) and low (DU‐145) expressing AR prostatic cancer cell lines used as positive and negative controls, respectively, exhibited similar patterns of AR receptor expression to the HTB‐131 and HTB‐132 cell lines, respectively. Incubation with DHT induced proliferation of AR‐expressing cells in a time‐dependent manner (Figure 3A).

Different concentrations of the peptide were used to evaluate the proliferation of AR‐expressing cells. Addition of the peptide alone had no effect without DHT, but the growth of HTB‐131 and HTB‐132 cells was substantially reduced (*p < .05, **p < .01, and ***p < .001) in the presence of DHT and increasing concentrations of the peptide (100–500 nM) (Figure 3). We used 200 nM of MPF as a negative control for the study, showing that it did not affect cell proliferation. However, the viability of prostatic cell lines with low AR expression (DU‐145) did not change substantially (Figure 3B). The peptide reduced cell viability of AR‐expressing cells in a dose‐dependent manner (Figure 3C).

### 3.2 Peptide increases caspase‐3 activity

**FIGURE 4:**
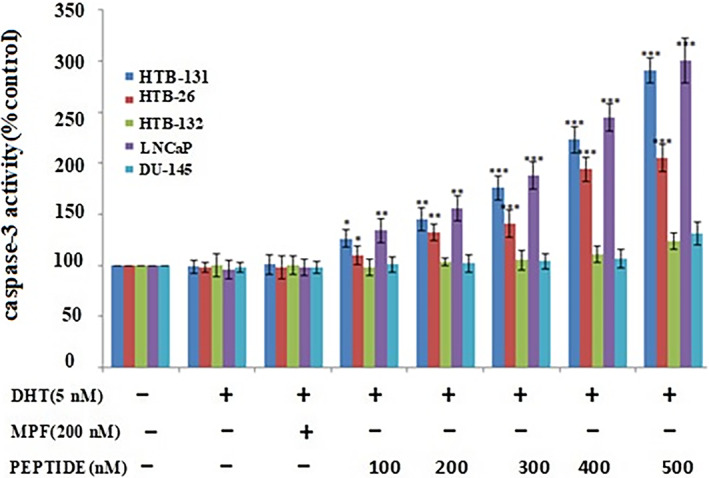
Caspase‐3 induction by the peptide in androgen‐expressing triple‐negative breast cancer cells. Cells were treated with various doses of the peptide for 24 h in the presence and absence of dihydrotestosterone. The peptide treatment increased the activity of caspase‐3 in androgen receptor‐expressing cells. MPF served as a negative control. Results (mean ± SD) were calculated as the percent of control values and a one‐way ANOVA analysis was performed. *p < .05; **p < .01; ***p < .001.

Various concentrations of the peptide were used to evaluate the pro‐apoptotic effect of the peptide in AR‐expressing cells. Addition of the peptide alone had no substantial effect in the absence of DHT; however, in the presence of DHT (100–500 nM), caspase‐3 was activated in a concentration‐dependent manner (*p < .05, **p < .01, and ***p < .001) (Figure 4). Caspase‐3 activity of HTB‐132 and DU‐145 cells were not affected (Figure 4).

### 3.3 The peptide increases Bax/Bcl‐2 ratio in AR‐expressing cell lines

**FIGURE 5:**
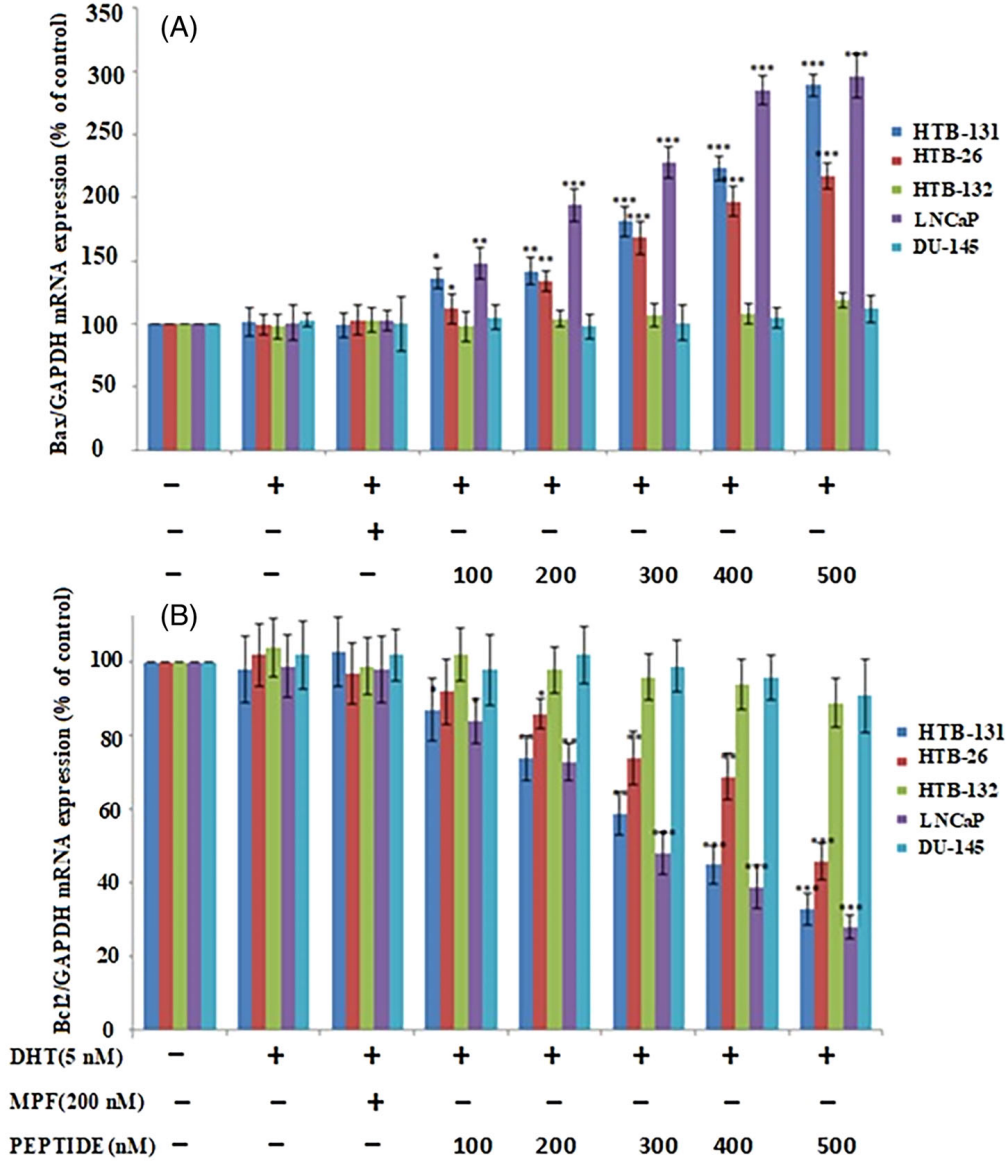
The effect of the peptide on the Bax/Bcl‐2 ratio of androgen receptor‐expressing triple‐negative breast cancer (TNBC) cells. Cells were incubated with different concentrations of the peptide for 24 h in the presence and absence of dihydrotestosterone. MPF was used as a negative control. peptide treatment increased the expression of Bax (A) and decreased the expression of Bcl‐2 (B) in androgen receptor‐expressing TNBC cells. Results (mean ± SD) were calculated as the percent of control values and a one‐way ANOVA analysis was performed. *p < .05; **p < .01; ***p < .001.

To further evaluate the pro‐apoptotic effects of the peptide, expression levels of Bax and Bcl‐2 mRNA were measured. Previous studies reported that Bax and Bcl‐2 play key roles in the intrinsic pathway of apoptosis which is regulated by mitochondria.^33^ The peptide treatment increased the expression of Bax and decreased the expression of Bcl‐2 in a concentration‐dependent manner (*p < .05, **p < .01, and ***p < .001, ANOVA) (Figure 5), resulting in increases in the ratio of Bax/Bcl‐2.

### 3.4 The peptide treatment increases apoptosis in AR‐expressing cell lines

**FIGURE 6:**
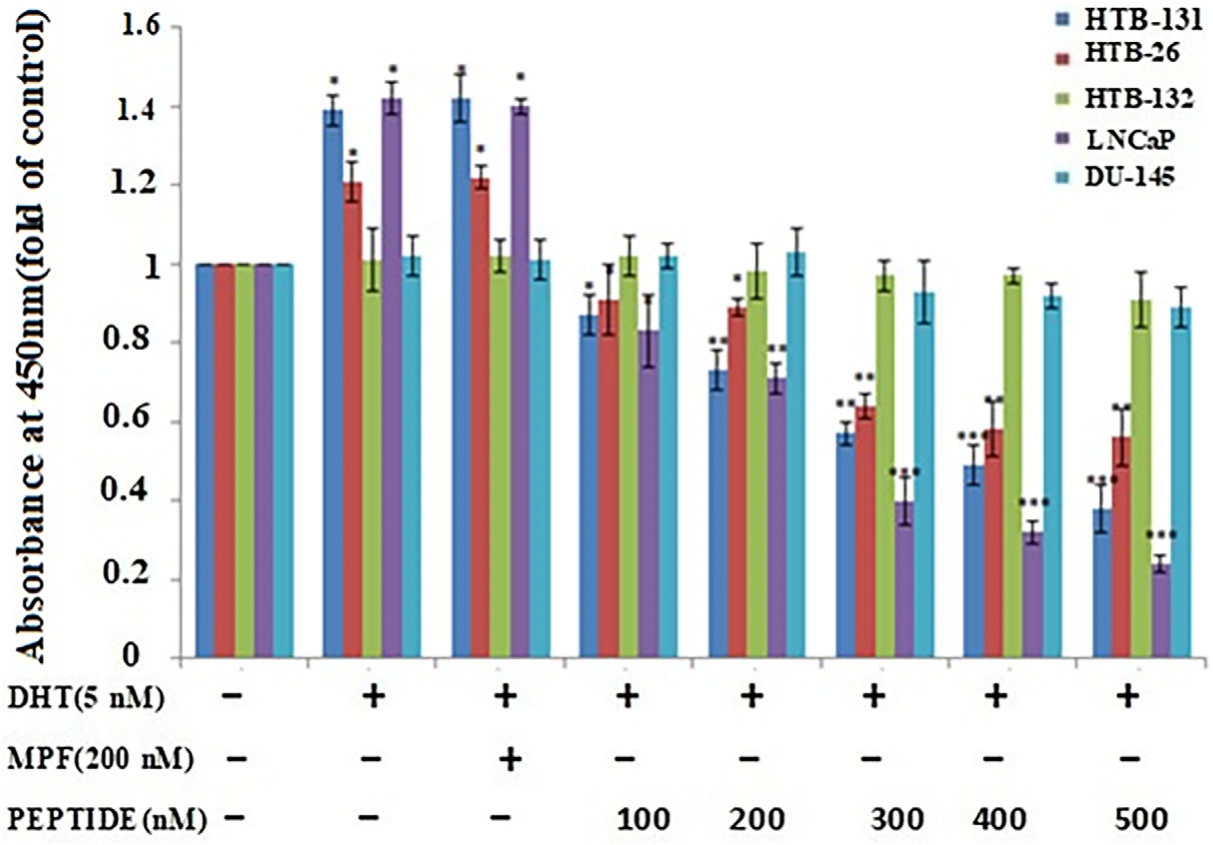
The effect of the peptide on BrdU incorporation in androgen receptor‐expressing triple‐negative breast cancer (TNBC) cells. Cells were treated with various concentrations of the peptide for 24 h in the presence and absence of dihydrotestosterone. The peptide treatment decreased the incorporation of BrdU in androgen receptor‐expressing TNBC cells. MPF was used as a negative control. Results (mean ± SD) were calculated as fold of control values. *p < .05; **p < .01; ***p < .001, ANOVA.

Flow cytometry can discriminate between cell apoptosis and necrosis, as well as changes in cell morphology and the presence of phosphatidylserine on the cell surface.^34^ We used BrdU and flow cytometry to further confirm the pro‐apoptotic effects of the peptide. Addition of the peptide alone did not affect BrdU incorporation, but increasing concentrations of the peptide (100–500 nM) in the presence of DHT reduced BrdU incorporation in a concentration‐dependent manner (*p < .05, **p < .01, and ***p < .001, one‐way ANOVA) (Figure 6).

**FIGURE 7:**
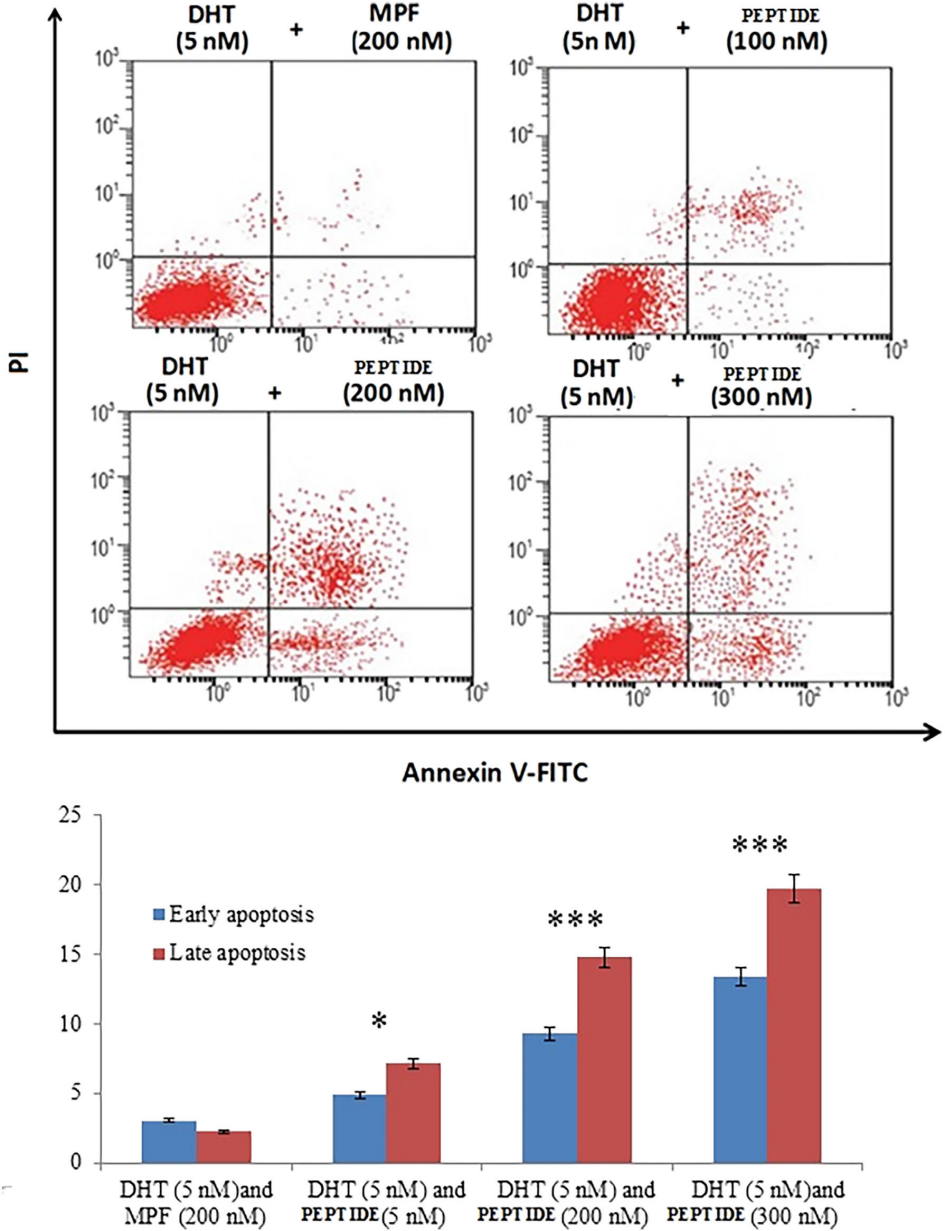
The effects of the peptide on early and late apoptosis induction in HTB‐131 cells. Cells were treated with various concentrations of the peptide for 24 h in the presence and absence of dihydrotestosterone. Early and late apoptotic cells were increased in response to the peptide treatment. MPF was used as a negative control. *p < .05; **p < .01; ***p < .001.

Additionally, the peptide incubation in the presence of DHT promoted early and late apoptosis in a concentration‐dependent manner (Figure 7).

## 4 DISCUSSION

The AR regulates proliferation, cell growth, and differentiation, and its role in the expansion and evolution of human cancers of importance.^35^ Emerging studies indicate that AR is overexpressed in TNBC,^33^, ^36^ and its expression levels correlate with tumor size, lymph node metastasis, and high‐grade tumors in patients with TNBC.^34^ These findings suggest that AR may be a potential target in the treatment of TNBC.^37^ Our findings show that a novel peptide (in the range of 100–500 nM) in the presence of DHT reduced growth of HTB‐131 and HTB‐26 cells in a concentration‐dependent manner. Prostatic cell lines with high and low AR expression levels (LNCaP and DU‐145) were used as negative and positive controls for AR.

To clarify the role of ARs on cell proliferation, studies by Cai et al. showed that activation of AR stimulated cell proliferation through upregulation of VEGF‐A, cyclin A, and cyclin D1 in HAECs.^38^ Moreover, androgens induce prostate cancer cell proliferation through the activation of mammalian target of rapamycin and post‐transcriptional increase of cyclin D protein.^39^

We first confirmed that AR is expressed in HTB‐131 and HTB‐26 cells as DHT increased their expression more than in HTB‐132 cells. Additionally, the peptide attenuated cell growth, caspase‐3 activation, and BrdU incorporation in the HTB‐131 and HTB‐26 cell lines. Treatment with concentrations of 100–500 nM of the peptide activated caspase‐3 and increased the Bax/Bcl‐2 ratio in a concentration‐dependent manner. The peptide (100–500 nM) in the presence of DHT reduced BrdU incorporation in a concentration‐dependent manner, and incubation with it reduced BrdU incorporation and promoted both early and late apoptosis. Further evaluation of this peptide showed that it specifically reduces cell growth in AR‐expressing cells, and that cells with minimal expression of AR (HTB‐132 and DU‐145) were partially resistant to the effects of the synthesized peptides.

In line with our study, Abazid et al. showed that when PC cells were treated with enzalutamide (an AR antagonist), the proliferation of PC cells was reduced in a time‐ and dose‐dependent manner. They also showed that in response to enzalutamide, the Bax/Bcl‐2 ratio was increased and genomic DNA was fragmented. Furthermore, enzalutamide affected the intracellular synthesis of steroid receptor‐associated heat shock proteins, thereby decreasing the expression of AR and ERβ1 proteins and inducing apoptotic pathways.^40^

Our findings indicate that DHT increases the proliferation of HTB‐131 cell lines more than in other cells. Moreover, DHT had the largest effect on HTB‐131 cell lines and the least effect on HTB‐132 cells. A study by Bryan et al. reported that the degree of tumor malignancy is higher in patients with low levels of ARs,^41^ while a study by Alshenawy indicated that AR positivity was associated with the occurrence of the disease, suggesting that low levels of AR expression were associated to with lower levels of malignancy.^42^

Several studies examined the effects of AR on the survival of TNBC patients or ER‐deficient patients.^43^ For instance, Agoff and colleagues did not discover a correlation between AR and patient survival in ER‐deficient patients.^44^ On the other hand, Gucalp et al. demonstrated a lack of AR in TNBC patients, while Jiang et al. attributed the presence of AR to an increased likelihood of disease recurrence.^45^

The mechanism of the pro‐apoptotic effects of this peptide is still unclear. However, Hsu et al. reported that the peptide inhibited AR transactivation,^26^ while Chen et al. reported that AR transactivation recruits c‐Jun and induces mitotic activation to increase proliferation.^46^ These findings indicate that the peptide treatment blocks AR transactivation and reduces cell growth. Similarly, Liao et al. reported that AR deubiquitylation induces cell suicide.^47^ To enhance the credibility of our findings, it would be beneficial to confirm the pro‐apoptotic effects of the peptide using in vivo models of cancer, such as TNBCs.

## 5 CONCLUSION

We used human breast cancer‐derived cells with high, low, and very low expression levels of AR to examine apoptosis caused by a novel peptide that targets ARs. Our study indicates that the peptide prevented the proliferation of breast cell lines in a specific manner. Cells with higher expression levels of AR were inhibited to a greater extent. For instance, cells with the highest expression of AR were more sensitive to the peptide, while cells lacking this receptor (HTB‐132 and DU‐145 cell lines) were unaffected. Similar findings were observed regarding the effects of the peptide on caspase 3, Bax, and Bcl‐2 proteins. Our results showed that the peptide altered cell division, decreased the expression of the Bcl‐2 gene and increased the expression of the Bax gene. Further in vivo studies, such as with solid TNBC tumors in animal models, are required to explore the therapeutic potential of the peptide.

## AUTHOR CONTRIBUTIONS

**Mazdak Jamshidi:** Data curation (lead); formal analysis (equal); funding acquisition (equal); investigation (equal); methodology (equal); resources (equal); software (equal); validation (equal). **Fatemeh Keshavarzi:** Conceptualization (supporting); investigation (equal); methodology (equal); project administration (equal); resources (equal); supervision (equal); validation (equal); visualization (equal); writing – original draft (equal); writing – review and editing (equal). **Sabrieh Amini:** Conceptualization (equal); data curation (equal); project administration (equal); supervision (equal); validation (equal); visualization (equal). **Ismail Laher:** Conceptualization (equal); funding acquisition (equal); resources (equal); validation (equal); visualization (equal); writing – original draft (equal); writing – review and editing (equal). **Ali Gheysarzadeh:** Conceptualization (equal); data curation (equal); formal analysis (equal); investigation (equal); methodology (equal); software (equal); writing – original draft (equal); writing – review and editing (equal). **Kambiz Davari:** Data curation (equal); methodology (equal); software (equal); validation (equal); visualization (equal).

## CONFLICT OF INTEREST STATEMENT

The authors declare that they have no competing interests.

## ETHICS STATEMENT

This assay did not include samples from humans or animals.

## Data Availability

The data used to support the findings of this study are included in the article.
